# Feasibility of a Multidomain Intervention Program for Community‐Dwelling Older Adults Based on Implementation Science (J‐MINT Community)

**DOI:** 10.1002/alz70860_102524

**Published:** 2025-12-23

**Authors:** Yujiro Kuroda, Kazuaki Uchida, Yoko Yokoyama, Ayaka Onoyama, Kosuke Fujita, Taiki Sugimoto, Hidenori Arai, Takashi Sakurai

**Affiliations:** ^1^ National Center for Geriatrics and Gerontology, Obu, Aichi, Japan; ^2^ Japan Multidomain Intervention Trial for Dementia Prevention Accreditation and Promotion Agency (JMAP), Obu, Aichi, Japan

## Abstract

**Background:**

Dementia poses serious challenges for individuals and communities. We first piloted a 12‐month multidomain program—encompassing exercise, nutrition, cognitive training, social engagement, and vascular risk management—in O City to assess feasibility. Building on these results, we then revised the intervention into a 6‐month basic course plus a 6‐month continuation class for H Town, aligning with municipal needs and the common practice of transitioning participants to self‐directed groups. We also trained local instructors and assessed a structured process for participant enrollment and follow‐up.

**Method:**

In 2023, we conducted a one‐arm trial in O City with 80 community‐dwelling older adults (aged 65–86). We defined feasibility as attendance at ≥60% of sessions over 12 months, and measured acceptability on a five‐point scale. Cognitive function was monitored via the Japanese version of the Montreal Cognitive Assessment (MoCA‐J; range 0–30), and food diversity was evaluated using a standardized questionnaire. In 2024, we implemented a revised format in H Town featuring a 6‐month basic program followed by a 6‐month continuation class. We employed a structured enrollment protocol comprising (1) identifying older adults with hypertension or hyperglycemia, (2) mailing invitations (*n* = 801), and (3) conducting an informational session. Three local instructors were trained, and we recorded participant enrollment and willingness to continue.

**Result:**

In O City, 75% of participants met the 12‐month attendance criterion, and the mean acceptability score was 4.4 out of 5. Mean MoCA‐J scores remained stable (22.8 ± 3.1 to 23.1 ± 3.5), while food diversity improved significantly (7.5 ± 2.8 to 8.4 ± 2.5; *p* = 0.008). In H Town, 33 older adults (4.1%) attended the informational session, and 19 enrolled. More than 70% indicated willingness to continue at personal cost after completing the revised program.

**Conclusion:**

Feasibility and acceptance of this multidomain dementia‐prevention program were demonstrated in two municipalities through distinct implementation models. Findings from O City informed the 6‐month structure in H Town, which aligns with local practices for transitioning participants to self‐directed groups. Future controlled studies should further clarify cognitive and health outcomes and refine recruitment and follow‐up strategies for broader community impact.

